# Asbestos-Related Disorders in Germany: Background, Politics, Incidence, Diagnostics and Compensation

**DOI:** 10.3390/ijerph15010143

**Published:** 2018-01-16

**Authors:** Xaver Baur

**Affiliations:** 1 European Society for Occupational and Environmental Medicine, EOM, Berlin, Germany; baur@eomsociety.org; 2 Emeritus, Institute for Occupational and Maritime Medicine, University of Hamburg, D-20246 Hamburg, Germany

**Keywords:** asbestos, asbestosis, mesothelioma, prevention, threshold limit values, compensation, stakeholders, asbestos ban, litigation

## Abstract

There was some limited use of asbestos at end of the 19th century in industrialized countries including Germany, but its consumption dramatically increased after World War II. The increase in use and exposure was followed by the discovery of high numbers of asbestos-related diseases with a mean latency period of about 38 years in Germany. The strong socio-political pressure from the asbestos industry, its affiliated scientists and physicians has successfully hindered regulatory measures and an asbestos ban for many years; a restrictive stance that is still being unravelled in compensation litigation. This national experience is compared with the situation in other industrialized countries and against the backdrop of the constant efforts of the WHO to eliminate asbestos-related diseases worldwide.

## 1. Introduction

Germany does not have any asbestos mines and 95% of the imports was as chrysotile asbestos from Canada and Russia. Industrial use began in 1871 at the Frankfurter Asbestwerke and in 1878 at the Sächsische Asbestfabrik in Radebeul. The first case of fatal asbestosis was a 35-year-old female worker at a German asbestos factory, published as early as 1914 [1]. As in other industrialized countries, the consumption of asbestos increased steadily in Germany, reaching a plateau of about 260,000 metric tons annually by the 1970s. There was a broad range of applications strongly dominated by the construction industry [2], without major differences in either use or regulations between the two German states that existed from 1946 until 1989.

The objective of this article is to provide a review of the historic background behind the pandemic tragedy of asbestos disease by focussing on the German experience. This will consider the contribution of the stakeholders and a sustained opposition to preventative regulations, the final establishment of a comprehensive asbestos policy and the resulting German asbestos ban in 1993. Even in spite of the well-known adverse effects of asbestos, preventative regulations were brought into force with a delay of decades, during which high levels of exposure persisted in the workplace. Further, a restricted compensation policy could be established, especially for asbestos-related lung cancer, without the need provide scientifically based justification.

### 1.1. Asbestos Consumption and Occupational Exposure in Germany

**Figure 1:**
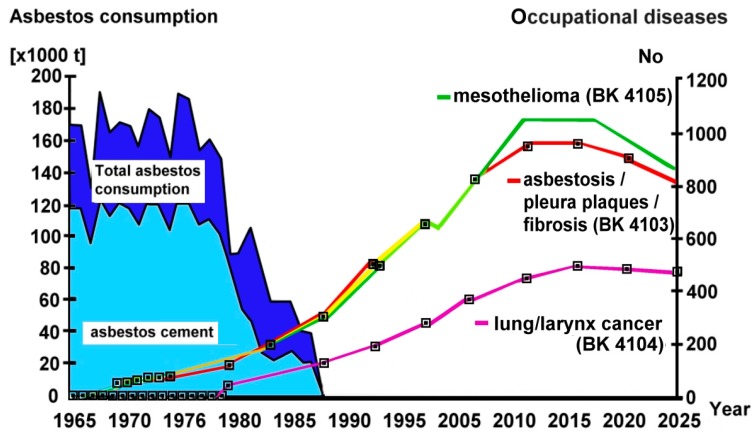
Asbestos consumption and recognized occupational diseases in West Germany (BRD), modified from [4]. In East Germany (DDR), the plateau of asbestos consumption was about 75,000 metric tons annually in 1975–1980. BK = occupational disease designation. Note, that past and current disease data were used for future estimates [4]. For evidence of underreporting of asbestos-related lung cancer see legend of Table 4.

In countries of the European Union after the asbestos ban in 2005, the use of asbestos has nearly ceased [3]. In Germany, there was a high rate of asbestos use in the 1960s and 1970s followed by a stepwise decline during the 1980s (Figure 1). Since the beginning of the 1990s and the asbestos ban in 1993, asbestos use has nearly disappeared, except for the persistence of about 80 metric tons used annually for the production of specific diaphragms.

### 1.2. Figures on Exposed Subjects and on Asbestos Exposure Levels

In industrial countries, 20 to 40% of adult men are thought to have held jobs that could have entailed some degree of asbestos exposure in the past few decades [5,6]. Such estimates are hampered, however, by the lack of reliable data on occupational and environmental exposure, especially for women. 

Exposure in the asbestos industry was usually high or extremely high up to the middle of the last century and declined in the subsequent decades in Western countries because of various preventative regulations and measures. Job exposure matrices based on exposure assessment are available for the USA, Finland, Germany, The Netherlands and Korea [6,7,8,9,10,11]. 

More than 26,000 measurements from mostly German workplaces or plants where asbestos was processed have demonstrated the greatest frequency of high exposure in the middle of the last century, in agreement with other industrialized countries. Exposure declined in the following decades, with the 90% percentile of asbestos fibre concentrations decreasing from 100/cm^3^ in the early 1950s to 40/cm^3^ in the early 1960s, to 10/cm^3^ in the 1970s and to 3/cm^3^ in the early 1980s in German factories [9,10,12]. For most workplaces, however, data on historical asbestos exposure is not known in detail. Based on the available measurements, therefore, exposure matrices have been developed to facilitate the assessment of health risk in decision-making processes [9,10,13]. 

### 1.3. Asbestos Threshold Limit Value

In Germany, a Threshold Limit Value (TLV) was enforced in 1973 and became more stringent in the following years and decades (Table 1). Initially, the TLV referred to weight (mg/m^3^) of fibres but later was changed to number of fibres per cm^3^ [9,10,14]. For details, see German Technical Rule for Hazardous Substances TRGS 519 [15] and *Gefahrstoffverordnung* [16,17], which nowadays takes EU regulations into account. 

The protection of workers and others who still work with asbestos and asbestos-containing hazardous materials, during demolition, reconstruction or maintenance work and waste disposal, is regulated by TRGS 519. This applies to activities involving asbestos-containing minerals, raw materials, preparations and articles manufactured from them. It establishes the general requirements for the protection of workers and others. Even when the asbestos fibre concentration in the workplace is low (<10,000 F/m³) because of applicable cancer risk preventative measures, vigilance in the reduction of asbestos fibres and their uptake is still needed. 

In addition to high prevalence in the workplace, household exposure from taking contaminated work clothes home also took place. Further, the general population has been exposed to asbestos fibres in the environment because of the widespread use of this material in building construction, pipes, filters, brakes, etc. Environmental fibre concentrations in urban locations have reached several hundreds fibres/m^3^ and up to 6000/m^3^ in buildings (IARC 2012). This non-occupational exposure can cause mesothelioma, which is still not covered by the German occupational disease regulations [2].

### 1.4. Medical Surveillance and Screening

Surveillance systems for asbestos workers have operated in many developed countries (e.g., in the USA, UK, New Zealand, Germany and Australia) by compulsory or voluntarily reporting systems that have provided informative lists of high risk occupations. 

In Germany, a mandatory registration and medical surveillance programme for former and current asbestos workers as well as a central registration of all of these workers was established in 1972 (ZAs; changing to Gesundheitsvorsorge Asbest, GVA, health examination for asbestos in 2008). This registry now comprises approximately 600,000 workers and, with the exception of a small group still engaged in destruction or maintenance, these are previously exposed subjects [2]. Approximately half of these workers were engaged in the construction industry. The reporting to the central register has been done by companies, insurance bodies, physicians and the workers themselves. There is some evidence that the number of previously exposed workers is significantly underestimated. 

The medical examination within the framework of this surveillance programme is performed by experts in occupational medicine; it consists of a medical and occupational history with special regard to respiratory complaints, physical examination, spirometry and chest X-ray. The examination is usually repeated at 2 to 3 years intervals. In the case of borderline findings or symptoms, a shorter interval until the next examination may be fixed and, if needed, a chest HRCT performed. If there is evidence for an asbestos-related disease then this must be reported to the statutory accident insurance institution (Arbeitsmedizinische Vorsorge nach der Verordnung zur arbeitsmedizinischen Vorsorge (ArbMedVV) Ministry of Labour and Social affairs; Arbeitsmedizinische Vorsorge G 1.2; DGUV; http://www.bmas.de/DE/Service/Medien/Publikationen/a453-arbeitsmedizinischen-vorsorge.html). 

### 1.5. Pressure on Recognition and Compensation of Asbestos-Related Diseases

Since lung cancer and mesothelioma were found to be associated with asbestos exposure in an increasing number of studies from the 1940s onwards [18,19,20,21], pressure from independent scientists, unions and governmental bodies arose not only for preventative measures but also for the recognition of and compensation for asbestos-related diseases (for details see the following section). These efforts finally resulted in stepwise inclusion of them in the lists of occupational diseases of various countries, including Germany.

Selikoff et al. [22,23] had already urged caution about the use of asbestos in the 1960s. In 1964, the New York Academy of Sciences held a historic course-changing conference (led by Selikoff) on the “Biological Effects of Asbestos” [24]. Selikoff also took part in three meetings in Germany on related topics and supported national initiatives on the prevention of asbestos exposure intensively: 1964 in Dresden, 1995 in Berlin and 1990 in Bad Reichenhall. Pressure for action arose from further studies in humans consistently confirming the relationship between asbestos exposure and mesothelioma as well as lung cancer [25,26,27,28,29,30,31,32].

The meta-analyses of 29 cohort studies encompassing 35 populations and of 15 case-control studies of asbestos exposure and laryngeal cancer undertaken by the Committee on Asbestos of the National Academy of Science and the Institute of Medicine (IOM) [33] demonstrated that there was sufficient evidence for asbestos causation for laryngeal cancer also. 

As presented in more detail in the Section 1.10 below, the asbestos industry systematically suppressed the release of early knowledge about the adverse health effects of asbestos [34]. This was true internationally as well as at national levels. However, legal discovery processes through civil lawsuits made this knowledge successively available [35]. The IARC [32], the Collegium Ramazzini [36] and the Joint Policy Committee of the Societies of Epidemiology (JPC-SE) [37] have repeatedly stated that all commercial types and all sizes of asbestos fibers are cancerogenic to humans.

### 1.6. Schedule of Asbestos-Related Diseases

#### 1.6.1. European Union 

Table 2 lists the current European schedule of asbestos-related occupational diseases, which does not have a legally binding status; for details see Guide to Diagnosis of the European Commission (Information notices on occupational diseases: a guide to diagnosis) [38]. Of most relevance to asbestos-related occupational diseases is asbestosis (301.21), mesothelioma following the inhalation of asbestos dust (301.22), complication of asbestosis in the form of bronchial cancer (302), fibrotic diseases of the pleura, with respiratory restriction, caused by asbestos (306) and lung cancer following the inhalation of asbestos dust (308). The pathologies of pleural effusion, diffuse pleural plaque or diffuse pleural thickening with (and without) respiratory restriction, rounded atelectasis of the pleura, and asbestos warts, are also mentioned but have not received an occupational disease designation. All aforementioned disorders are listed in the Guide to Diagnosis of the European Commission (Information notices on occupational diseases: a guide to diagnosis). Furthermore, cancer of the larynx, cancer of the trachea and cancer of the ovary are diseases for which the causal link is exposure to asbestos dust, are also well established and it is generally agreed [32,39] that they will be included in the forthcoming update of the occupational diseases list. This may also applys to cancer of the pharynx, cancer of the stomach, cancer of the colon and rectum, although generally not showing significant positive associations with asbestos exposure. Possible associations have also to be taken into consideration by EU funded research in future to help clarify whether the current limited evidence can be strengthened. 

#### 1.6.2. History of the List of Asbestos-Related Diseases in Germany 

As described by Robert N. Proctor in his book *The Nazi War on Cancer* [40], physicians in Germany had already documented the health hazards of asbestos in the 1930s. Asbestosis was included in the list of occupational diseases in 1936 and by 1942 Germany became the first nation to recognize lung cancer caused by asbestos inhalation as an occupational disease worthy of compensation (Table 3a). Already at that time, a wide range of public health measures was passed including, among others, aggressive anti-smoking campaigns, restrictions on asbestos use, as well as on radiation, pesticides and food dyes. Nazi health officials introduced strict occupational health and safety standards; however, the perverted Nazi ideology intended that only the “nordic race” was to be protected from these hazards. 

More recently, in addition to mesothelioma and cancer of the lung and larynx [41,42,43], pleural disorders as well as lung and laryngeal cancer in combination with pleural disorders that have arisen from asbestos and/or an asbestos dose of at least 25 fibre-years have also become occupational diseases [44]. A recommendation to include cancer of the ovary, for which a meta-analysis identified nearly a doubled risk (The doubling of a disease risk through an occupation above that which occurs in the general population is the applied threshold for inclusion of a disease in the German list of occupational diseases) in asbestos-exposed females [45] has been made. The prevailing German legal definitions of the four asbestos-related occupational diseases and their designations are shown in Table 3b.

The limited evidence for asbestos causation of colorectal cancer, cancers of the stomach [46] and pharynx [32,33] means that these disorders have not been acknowledged and are not compensated.

### 1.7. German Guideline on Diagnosis of and Compensation for Asbestos-Related Diseases

**Figure 2:**
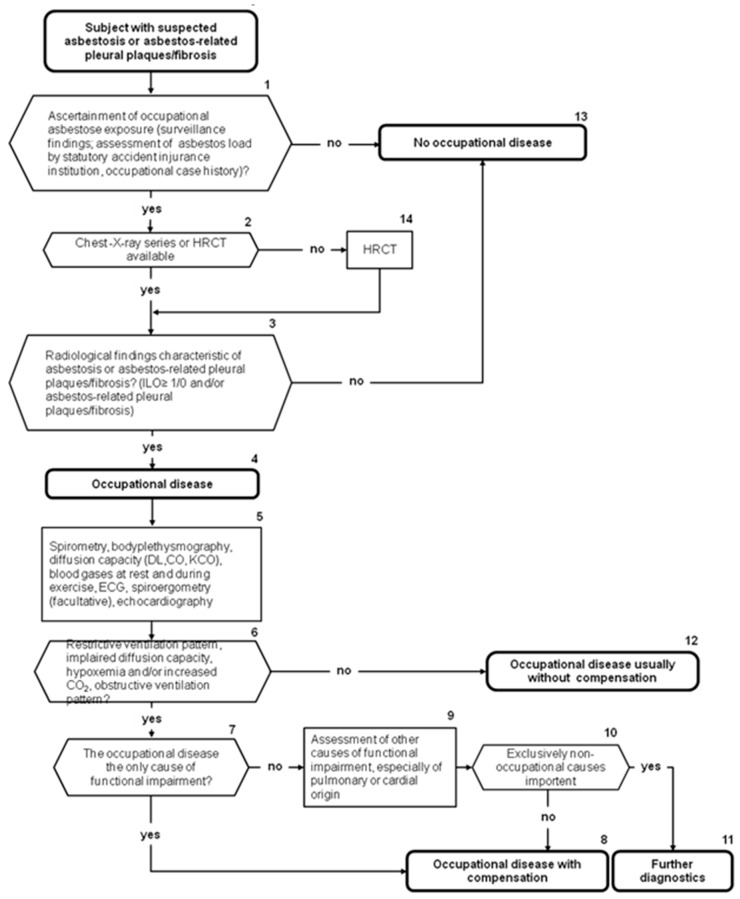
Algorithm of medical expert examination for the diagnosis of asbestosis or asbestos-related pleural plaques/fibrosis according to the German guideline “Diagnostics and expert opinion of asbestos-related occupational diseases”.

In 2011 the first version of the guideline on diagnostics and compensation of asbestos-related diseases was issued [47] (currently it is being updated). Figure 2 shows the recommended stepwise diagnostic approaches with regard to asbestosis and asbestos-related pleural plaques or fibrosis. Histopathological confirmation is needed for suspected asbestos-related malignancies and for the resolution of differential diagnoses but not for the diagnosis of non-malignant asbestos-related disorders. In any case the diagnosis is based on a detailed exposure assessment, occupational and medical case history, an appropriate latency, the clinical picture and symptoms, and radiological and lung physiology findings. 

In addition to the occupational history, a chest X-ray is fundamental for identifying asbestos-related diseases, although high resolution computed tomography (HRCT) is recommended in all doubtful situations because of its much higher sensitivity and specificity [48]. 

The recommendations concerning the degree of compensation for occupational diseases considers the disease-related impairment of the individual in relation to the working market; details are given in the guideline for diagnostics and compensation of asbestos-related diseases [47].

### 1.8. Asbestos-Related Diseases in Germany

After a mean latency period of malignant disorders of about 38 years, a rapid increase in acknowledged asbestos-related diseases occurred, while in more recent years a plateau has been reached, Figure 1. Mean asbestos exposure duration was 18 to 20 years and the mean age of diseased workers is 67 years. Mesothelioma, lung cancer and less frequently asbestosis are important causes of occupational disease mortality (about 62% of all deaths from occupational diseases) [2]. 

The total number of reports of asbestos-related diseases is in the range of 9400 annually of which about 4100 (45%) are acknowledged as occupational diseases and about 2500 (25%) receive compensation. In respect of individual diagnoses, between 80% (mesothelioma) and 20% (lung cancer) of reported cases are subsequently acknowledged (for details see Table 4). There is, for example, a discrepancy between the reported and acknowledged numbers for asbestos-related lung cancer. There is evidence that asbestos-related lung cancer, especially, and asbestosis are significantly underreported; a major reason for this is the overreliance on unsound lung fibre counts (see Section 1.10, below). 

### 1.9. Health and the Economic Burden on Society

The asbestos cancer pandemic is estimated to cause 100,000 deaths annually in western countries. More than 10 million deaths will have occurred globally, from before when asbestos was banned worldwide and after all exposure has ended [53,54,55]. Estimates for Europe are 20,000 cases of lung cancer and 10,000 cases of mesothelioma per year [54], i.e., 5–7% of all cancer cases might be attributable to occupational asbestos exposure [55]. It can be assumed that the total number of asbestos-related deaths (including asbestosis cases) is at least double these figures [56] because of long latency periods leading to large number of underreported cases. The asbestos-caused disorders, especially asbestosis, considered benign are responsible for much greater rates of morbidity and are frequently associated with premature death. It should be mentioned, however, that the current global production of asbestos continues at a high level, which means that these figures may not reflect the true ultimate burden of the pandemic. Estimates of attributable fractions in the population were made, among others, in the UK [57], although these are highly time and place specific. 

In parallel with the increase in numbers of asbestos-related diseases, there was a steady increase in associated costs [58]. Table 5 presents estimates of costs in 2009 and 2012 for mesothelioma alone for 15 European countries. Germany is ranked in fourth place and current expenses for all acknowledged asbestos-related diseases in Germany are more than €500 million annually. More than 80% [59] has to be spent on pensions for asbestos victims or their dependents and about 17% on medical rehabilitation [2]. These costs do not include disorders arising from environmental asbestos exposure nor from asbestos removal.

In Germany, the potential years of life lost, attributable to asbestos exposure, amounted to more than 150,000 in the period 1992–2002 and to around 20,000 annually, subsequently [2].

### 1.10. Role of Stakeholders—Their Socio-Political Contributionsand Influence on Policy-Making Processes

In the 1970s, the Asbestos International Association (AIA) was founded in order to promote the interests of the asbestos industry internationally. It created an effective well-coordinated network for global operations concerned with asbestos promotion, manufacturer and processing industries, with the express intention of promoting worldwide asbestos consumption (AIA 1970). Its successor, the International Chrysotile Association [60], is still active and continues to spread the unfounded thesis that certain types of asbestos are safe to use [61].

The asbestos industry could even influence policy-making processes at the European level and this was a major reason why the EU considered asbestos to be safe to use for so long. It was not until 2005 that a general asbestos ban was brought into force in the European Union.

The successful lobbying by the international (AIA) and national asbestos industry (with Eternit owned by Schmidheiny a leading proponent in Germany) succeeded in doubling asbestos consumption in the 1960s and 1970s, in Germany and in many other countries, in spite of the increasing knowledge on the health hazards of asbestos since the middle of the last century. The asbestos industry ignored all the hazards of asbestos, kept quiet about it, played it down and prevented the listing of asbestos in risk category I (“very hazardous”) in the German hazardous materials regulations and of asbestos-containing products being labelled as carcinogenic until 1996. They also provided 2.5 million German Marks of funding to the Federal Health Agency (BGA) of the German state for investigations that strongly supported them. Remarkably, the BGA often voted to protect the economic interest of the asbestos industry rather than in favour of health protection, as did the so-called “Independent Advisory Committee for the Asbestos Industry“ (chaired by Prof Valentin, one of the most influential university occupational medics). The industry started a war against the ever-increasing data on asbestos-related diseases and, more specifically, against independent researchers, such as Prof. D. Henschler, Prof. H.-J. Woitowitz, Prof. K. Norpoth, Prof. I.J. Selikoff, the Federal Environmental Agency (UBA) as well as against a major trade union (IG Metall), which warned of the health “time bomb” of asbestos [62]. It should be mentioned that not all German trade unions adopted an anti-asbestos position, since many workers were afraid of losing their jobs. The asbestos industry sponsored scientific articles and initiated ghostwriting for publications in toxicology journals. This coordinated “product defence” was designed to seed the literature with strategic science so that liability claims by asbestos victims could be defeated [63,64,65]. The annual report of 1979 by the German Trade Association Asbestos e.V. (Wirtschaftsverband Asbest e.V.) documents the propaganda of the asbestos lobby and its contining efforts to disinform ministries, state labor inspectorates and social accident insurance associations, by claiming that any bans or categoric substitution orders on the use of asbestos would represent a substantial risk for the economy, the asbestos industry and many jobs.

As a result of these controversial socio-political influences, only non-binding initial recommendations were issued by the accident insurance association in the early 1960s that were designed to control the adverse health effects of asbestos. A first technical guideline concentration (TRK value) for asbestos was scientifically justified and published in 1973. Although there were some more strict regulations in the following years, it was not until the 1980s that state bodies and the statutory accident insurance associations started to react in a significant way [62]. 

As an example of successful trade union and worker activities, the situation in the Bremer Vulkan shipyard is worth mentioning. Already in the mid 1970s, in opposition to some of the unions and the Social Democratic Party, the workers council and the left-wing “Echolot” workers group resisted the reconstruction of ships heavily contaminated with asbestos, started information campaigns and introduced preventative measures previously neglected [66].

Increasing public interest, publications in the media and socio-political discussion eventually resulted in a cumulative general awareness and contributed to a decrease in asbestos consumption. A scandal in one of the asbestos companies in the early 1980s, for example, where more than 100 asbestos-related deaths became known in the company, resulted in closure of the site. The equipment was simply exported to South Korea and production continued.

Compensation for occupational disease reached 4104 individuals in 1993. Lung and laryngeal cancer caused by asbestos was incorporated with the assistance of I.J. Selikoff and team when an asbestos load of 25 fibre-years become accepted as a sufficient precondition, resulting in a significant increase in the number of recognized cases. This was turned down after the Mesothelioma Register, owned and paid for by the statutory accident insurance institutions and after performing many thousands of expert analyses of their monopolistic lung examinations on behalf of the accident insurance institutions, introduced a new restrictive histological definition of the disease. This did not follow the CAP/NIOSH definition [67] and relied more on that of Roggli, who had been supported by the US asbestos industry [68]. The Mesothelioma Register definition reinterprets the initial pathological changes of grade 1 asbestosis, according to CAP/NIOSH, as grade 0 [69]. Furthermore, and most importantly without any scientific evidence, they defined the presence of a certain number of asbestos fibres and asbestos bodies in lung tissue as a pathohistological precondition for asbestosis and asbestos-related lung cancer [70,71,72]. This latter requirement is especially inappropriate for chrysotile because of its short half-life in the lung and its inability to form asbestos bodies. Subsequently, the number of accepted lung cancer cases has plateaued at approximately 800 per year despite the strong linear increase in reported cases (see Figure 1).

### 1.11. Factors Initiating Improvement of Working Conditions and a Definitive Asbestos Ban

Asbestos-related diseases peak after a mean latency period of 38 years in Germany. The official statistics indicates more than 4000 new cases with 1600 deaths, an estimate of 20,000 potential years of life lost, and €500 million expenses annually. There is evidence that underreporting and underdiagnosis is substantial and that the real figures are much higher. This has elicited concern among exposed workers and controversies in policy-making boards since the 1970s, as the asbestos industry has continued to ignore or even deny the harm caused by asbestos. However, independent physicians and scientists as well as unions continued to highlight feasible and effective preventative measures and ethical principles. This was the driving force behind the establishment of job exposure matrices, the introduction of new regulations reducing stepwise exposure limit values, the inauguration of appropriate occupational diseases and, ultimately, for the establishment of a definitive asbestos ban in 1993. Further motivation for this development was the recognition of the enormous financial burden created by asbestos-related diseases.

Hopefully, countries still mining and using asbestos will take the data into account that shows the many thousands of suffering individuals and associated costs and decide to take action to minimize the asbestos-related burden of their own societies in future. 

## 2. Discussion

### Lessons Learnt from the Burden of Asbestos-Related Disorders and Deaths

#### Past Pandemic and Current Battlefields

The major lesson from the profound worldwide asbestos tragedy for today and the future is that the asbestos-related diseases and deaths were totally preventable. However, the economic interests of a powerful industrial group prevented timely appropriate political decisions and binding regulations from being made, nationally, in Europe and also worldwide.

The asbestos tragedy was allowed to happen in spite of the clear association between asbestos exposure and numerous illnesses that was substantiated by an overwhelming body of evidence. It took decades of socio-political controversy before the use of asbestos was successively restricted or prohibited in most western countries. This delay was partially due to a lack of research on asbestos-caused disorders, but mainly from a reluctance by both industry and governmental authorities carefully orchestrated by the intensive and successful lobbyism of the asbestos industry (which remains active in some asbestos-exporting countries, including Russia, China, Brazil, Zimbawe, Kasakhstan [73]), and even corruption [74,75]. Nowadays, the asbestos industry is focussing on saving the reputation of chrysotile asbestos by promoting the false notion that it is safer than other forms of asbestos. A recent example of this is the report by Bernstein [76] on “The health risks of chrysotile asbestos” in animals that was sponsored by the asbestos industry. This publication has selected literature and scientifically flawed clinical and scientific knowledge, based mainly on their own published animal studies, frequently with a follow-up of only 90 days. The author fuels the propaganda by claiming that “the studies of chrysotile cement workers clearly demonstrate that under controlled use of chrysotile, it can be used safely”. This opinion is in marked contrast with the rest of the available literature that provides highly significant evidence to the contrary. The list showing adverse health effects from chrysotile asbestos in humans as well as in animal studies is extremely long. Based on this evidence, the International Agency for Research on Cancer, IARC, has also classified chrysotile asbestos as a group 1 carcinogen [77,78]. For decades, IARC and WHO have maintained that there is no exposure to well known carcinogens with a corresponding risk of zero. This is even more the case for asbestos, in which short exposure to relatively low concentrations has been shown to cause mesothelioma. 

Out of nearly 200 counties, 55 have banned the use of asbestos, specifically the import, export, trade and manufacture of asbestos and asbestos containing material. The Scandinavian countries had already banned the use of asbestos in the mid-1980s, Germany in 1993, France in 1996 and the UK in 1999. The European Union has released several directives regulating work with asbestos [79] since the 1980s and issued a ban directive that took effect in 2005 [80]. In spite of banning asbestos, some countries still import asbestos (e.g., Germany with 80 Mt annually) by applying EU regulation exemptions [81].

In contrast, at the 8th Conference of the Parties to the Rotterdam Convention in Geneva in May 2017, Russia, Kazakhstan, Zimbabwe, India, Kyrgyzstan, Belarus and Syria refused to allow chrysotile asbestos to be added to the Convention list of hazardous substances. The Rotterdam Convention requires countries to obtain Prior Informed Consent from any country to which they wish to export a hazardous substance on the Convention list. The decision to add a hazardous substance to the list is made by consensus. For more than a decade, a handful of countries that profit from the asbestos trade have blocked a consensus and, thus, sabotaged the Convention and its recommendations.

The ICA, whose role is to promote the continued use of chrysotile asbestos, has again heavily lobbied against the listing of chrysotile asbestos as a hazardous substance under the Rotterdam Convention [60,82].

## 3. What Still Has to Be Done

Firstly, a worldwide ban of mining and use of all types of asbestos is urgently needed. However, because of the long latency period of asbestos-related diseases, such bans must be accompanied by appropriate preventative strategies, as a ban alone will not result in immediate elimination of these disorders. If the global use of asbestos were to cease today, a decrease in the incidence of asbestos-related diseases would only become evident in approximately 20 years [36,83].

It has to be emphasized that in most European countries, including Germany, the asbestos ban has not been followed by proper cleaning up of the asbestos-containing materials that were present at the time of the ban and/or are still present. The European parliament approved a resolution in 2013 declaring 2028 as a reasonable deadline for an asbestos-free Europe. This remote date provides an indication of the severity of the situation and the lack of interest by governments.

In conclusion, it is obvious that there is an urgent need for closer international cooperation in preventative strategies, the banning of all mining and the use of asbestos. The health risks of exposure to asbestos cannot be controlled by technology or by regulation of work practices. Safe or at least safer substitutes for asbestos are available and should be used exclusively [84,85]. The failure of a timely and effective preventative management of asbestos, because of the strong influence of an interest group and its affiliates, including scientists and physicians, must lead us to take a dismal view of the preventative regulations currently being pursued for the more than one hundred other carcinogenic substances used in the workplace. 

### Key Notes

- Distribution of scientific knowledge on the harm of asbestos and the related enormous economic burden on society has increased the concern of exposed workers and the public.
- This has forced policymakers to initiate regulations on effective preventative measures and compensation for asbestos victims.
- Latency periods until appropriate regulations and an asbestos ban come into force are associated with many new asbestos-related diseases and, therefore, such latency periods should be minimized.
- Fair compensation for asbestos victims is needed.
- Alternative harmless products that replace asbestos should be promoted.
- Expensive cleaning up of asbestos-containing materials, e.g., in buildings, has to be taken into account.

## 4. Conclusions

As expected the strong increase in use of asbestos in the middle of the last century was followed by high numbers of asbestos-related diseases and a high economic burden on the society. Although the causal relation between asbestos exposure and these diseases have been well-known it can be recognised that corporate interests are able to hinder appropriate regulatory and preventive measures and a timely asbestos ban, and frequently also fair compensation of asbestos victims. Countries still mining and using asbestos should take into consideration these experiences including the worldwide human asbestos tragedy and the enormous cost forthcoming in the public health sector. Additional cost will follow due to needed proper cleaning up of asbestos-containing material, especially in buildings.

## Figures and Tables

**Table 1:** Stepwise reduction of the German asbestos threshold limit value (TLV)

Implementation (Year)	Fibre Type	TLV (mg/m^3^), fibres/m^3^
1973	Chrysotile *	(0.15)
1976	Chrysotile *	2 × 10^6^; (0.10)
1979	asbestos (chrysotile, crocidolite, amosite)	10^6^; (0.05)
1985	crocidolite	5 × 10^5^; (0.025)
1990	chrysotile, crocidolite, amosite	2.5 × 10^5^
1996	asbestos (chrysotile, crocidolite, amosite)	no TLV 15 × 10^5^ fibres/m^3^ initiate preventative measures for sanitation workers (personal safety medical surveillance programme)
2014	asbestos (chrysotile, crocidolite, amosite)	no TLV 10 × 10^5^ fibres/m^3^ initiate preventative measures for sanitation workers (personal safety medical surveillance programme)

* Chrysotile was the predominant type of asbestos used in Germany (about 95%).

**Table 2:** The European schedule of occupational diseases.

L 238/30	Official Journal of the European Union.9/25/2003 ANNEX I
3	Diseases caused by the inhalation of substances and agents not included under other headings
301	Diseases of the respiratory system and cancers
301.21	Asbestosis
301.22	Mesothelioma following the inhalation of asbestos dust
301.31	Pneumoconioses caused by dusts of silicates
302	Complication of asbestos in the form of bronchial cancer

**Table:** 

**(a)**	
**Occupational Disease**	**Year**
Asbestosis	1936
Lung cancer in connection with asbestosis	1942
Mesothelioma of pleura and/or peritoneum	1977
Asbestosis or asbestos-related pleural plaques or fibrosis	1988
Lung cancer combined with asbestosis or asbestos-related pleural plaques or fibrosis	1988
Lung cancer combined with asbestosis, asbestos-related pleural plaques or fibrosis or evidence of cumulative exposure to asbestos dust in the workplace of at least 25 asbestos fiber-years	1992
Larynx cancer combined with asbestosis, asbestos-related pleural plaques or fibrosis or evidence of cumulative exposure to asbestos dust in the workplace of at least 25 asbestos fiber-years	1997

**Table:** 

**(b)**	
**Occupational Disease No.**	
4103	Asbestosis or diseases of the pleura (plaques, fibrosis) caused by asbestos dust
4104	Lung or larynx cancer * - combined with asbestosis - combined with diseases of the pleura caused by asbestos dust or - if there is evidence of a cumulative exposure to asbestos dust in the workplace of at least 25 fiber-years {25 × 10^6^ [(fiber/m^3^) × years]}
4105	Mesothelioma of the pleura, the peritoneum or the pericardium caused by asbestos
4111	Lung cancer caused by the interaction of asbestos dust and polycyclic aromatic hydrocarbons, by evidence of exposure to a cumulative dose which equates a probability of causation of at least 50 percent

* cancer of the ovary will be also included in future and, currently, acknowledgement and compensation of asbestos-related ovary cancer is already possible according to paragraph 9, Section 2, of the German Social Law VII (Sozialgesetzbuch VII); according to this section a disease can also be acknowledged and compensated for, even when not on the list of occupational diseases, when new scientific knowledge clearly documents an occupational cause and the other legal preconditions of an occupational disease are fulfilled.

**Table 4:** Number of asbestos-related disorders officially recognized as occupational diseases in Germany 2013–2016; 2013–2015 data from [49], 2016 data from BK-Dok [50].

Asbestos-Induced Occupational Diseases (OD) in Germany in 2016, 2015, 2014, 2013					
Occupational Diseases No.	Reported OD	Acknowledged OD	Newly Compensated OD	Deaths	Year
4103 Asbestosis, Pleural fibrosis/plaques	3607	2183	578	166	2016
	3712	2002	541	165	2015
	3602	1997	603	153	2014
	3636	1926	582	159	2013
4104 Lung or larynx cancer	4368	912	814	618	2016
	4482	773	715	593	2015
	4343	834	766	595	2014
	4079	794	711	559	2013
4105 Mesothelioma	1304	1031	944	857	2016
	1417	958	881	812	2015
	1380	1048	976	817	2014
	1425	978	904	734	2013
4114 Lung cancers due to asbestos + PAH	125	22	19	15	2016
	138	33	28	11	2015
	132	23	20	18	2014
	142	24	24	17	2013
Total (2016)	9404	4148	2353	1656	

Note: There is evidence for significant underreporting especially of asbestos-related lung cancer since the ratio between this disorder and mesothelioma is typically in the range of 3.0 [51,52].

**Table 5:** Estimates of mesothelioma costs of 15 European countries [59].

Country	Number of Mesothelioma Cases	Costs 2009 ^a^ (€)	Number of Lung Cancer Cases	Costs 2012 ^b^ (€)
Austria	80	10,000,000	160	487,001,280
Belgium	156	19,500,000	2512	7,645,920,096
Denmark	71	8,875,000	142	432,213,636
Finland	75	9,375,000	150	456,563,700
France	826	103,250,000	1652	5,028,288,216
Germany	1063	132,875,000	2126	6,471,029,508
Italy	1235	15,437,500	2470	7,518,082,260
The Netherlands	395	49,375,000	790	2,404,568,820
Norway	54	6,750,000	108	328,725,864
Poland	96	12,000,000	192	584,401,536
Portugal	19	2,375,000	38	115,662,804
Romania	58	7,250,000	116	353,075,928
Span	263	32,875,000	526	1,601,016,708
Sweden	123	15,375,000	246	748,764,468
United Kingdom	1891	236,375,000	3782	11,511,492,756

^a^ Based on the €125,000 estimated cost of one average case of mesothelioma in France. The French figures are atypical and appear to exclude several elements of a fully costed case of ARDs. ^b^ Based on the €3,043,758 average cost of a case of lung cancer due to chemical exposures estimated under REACH. The REACH figures for a typical case of occupational cancer are more comprehensive and include elements for pain and suffering.
